# Focal Treatment of Prostate Cancer with Vascular-Targeted Photodynamic Therapy

**DOI:** 10.1100/tsw.2008.127

**Published:** 2008-10-03

**Authors:** Scott E. Eggener, Jonathan A. Coleman

**Affiliations:** Urology Service, Department of Surgery, Memorial Sloan-Kettering Cancer Center, New York, USA

**Keywords:** prostate cancer, focal therapy, photodynamic therapy

## Abstract

Epidemiologic and pathologic features of prostate cancer have given rise to an interest in focal treatment for carefully selected patients. Prostate cancer remains highly prevalent, particularly in the U.S. and Europe. As screening programs have become more aggressive and widespread, a substantial proportion of men diagnosed with localized prostate cancer have disease characteristics associated with a low risk of progression. Treatments such as radical prostatectomy and radiation therapy can lead to durable recurrence-free survival in most patients, but carry variable risks of bowel, urinary, and sexual side effects. Few men and few urologists are comfortable leaving a potentially curable prostate cancer untreated. Focal therapy offers an attractive alternative for the patient faced with a choice between aggressive local intervention (radiation or surgery) and watchful waiting. Contemporary diagnostic biopsy strategies and imaging tools, and the development of predictive statistical models (nomograms), have led to improvements in tumor characterization and risk stratification, making focal therapy a viable treatment option for specific men. This article reviews the rationale and indications for focal therapy and highlights vascular-targeted photodynamic therapy (PDT) as one of many promising focal therapy techniques.

## WORLDWIDE HEALTH PROBLEM

Prostate cancer continues to be a frequent cause of morbidity and death throughout the world. Despite the formidable prevalence of prostate cancer, no consensus exists regarding the merits of screening, selection of patients for primary treatment, and optimal treatment modality. The impact of disease and treatment-related morbidity has wide-ranging public health implications, particularly in countries where screening is common and male life expectancies are increasing.

In 2004, an estimated 68,000 men in the European Union died of prostate cancer, making it the third most common cause of cancer-related death[1]. In the U.S., 27,000 men were projected to die of prostate cancer in 2007, continuing a trend of declining mortality over the past decade[2]. While the disease burden in East Asian countries, such as Japan, remains lower than in Europe and the U.S., age-standardized mortality rates have been dramatically and continuously rising over the past 40 years[3].

## PROSTATE CANCER DETECTION

Prostate specific antigen (PSA) testing was approved in 1986 by the U.S. Food and Drug Administration to monitor men with prostate cancer. By the time PSA testing was formally approved for cancer detection in 1994, PSA screening had already become widespread, effectively ushering in the contemporary era of prostate cancer detection. Consequently, the age-adjusted incidence of prostate cancer has changed dramatically over the past several decades, rising from less than 92 cases/100,000 men in 1975 to a peak of 240 cases/100,000 men in 1992, before declining to a relatively constant level of 180 cases/100,000 men since 2001[4]. Since 1994, annual age-adjusted mortality rates in the U.S. have also steadily decreased, probably but not certainly as a result of early detection and effective therapy of potentially lethal early-stage cancers.

Despite the shift toward improved prostate cancer detection and early diagnosis seen in the last 2 decades, the absence of an equally large impact on prostate cancer mortality has led some to question the benefits of PSA screening strategies. For example, in the U.S., the estimated ratio of incidence to mortality in 2006 was 8.6[5]. In contrast, PSA screening has not been widely embraced in Europe. The lower incidence-to-mortality ratio in Europe (e.g., approximately 3.0 in the U.K.) suggests a comparatively lower rate of early diagnosis[6].

The discrepancy in incidence to mortality is likely due, in part, to increased detection of slow-growing or relatively indolent prostate tumors among highly screened patients. Indeed, there is evidence to support a relatively high prevalence of indolent tumors that pose little immediate risk to health and life. Autopsy studies have shown high rates of incidental, localized, moderately differentiated, prostate cancer in men who died from other causes. Prostate cancer has been identified in 25–30% of these men[7], with some studies reporting rates as high as 29% for men in their thirties, 32% for men in their forties, 55% for men in their fifties, and 64% for men in their sixties[8]. Thus, prostate cancer screening is likely to identify many cancers that would not have shortened the lifespan of many men, as suggested by the seemingly opposed trends of increased clinical detection[4], but decreased autopsy detection of prostate cancer in the PSA era (a nearly threefold change from the 1950s to 1990s[7]).

Despite the geographic variations in prostate cancer screening, and the purported benefit of screening on early detection and disease-specific survival, the weight of these observations suggests that most men diagnosed with low-volume, moderately differentiated, prostate cancer will not succumb to this disease during their lifetime. The public health, individual, and financial implications are profound. It is admittedly difficult to predict with certainty an individual patient's long-term risk based on currently available pretreatment variables and trends of increasing life expectancy. However, by any metric, a substantial population of men is overdiagnosed and overtreated.

## THE TREATMENT OF PROSTATE CANCER AND ITS IMPLICATIONS

At the time of diagnosis of a low-risk prostate cancer, a man may choose active therapy or watchful waiting, more recently referred to as active surveillance. For many men, foregoing treatment initially is an acceptable alternative. In avoiding the morbidity of treatment, they accept the rare but real possibility that the cancer will progress and become less curable at a later time. An active surveillance program attempts to minimize this risk by requiring scheduled office visits with repeat biopsy sessions at predetermined intervals or when clinically indicated. Active treatment may be recommended when there is evidence to suggest disease progression. Many criteria have been advanced as a threshold for intervention, but none have been validated as effective. Patient anxiety associated with these programs leads many to abandon this strategy despite a favorable clinical course, and, conversely, a small but meaningful number of men later found to have high-risk disease may be ill served by delaying treatment[9].

In the U.S., the health care community generally recommends active treatment with curative intent for healthy men with localized prostate cancer[10]. This approach aims to limit the number of men dying of prostate cancer at the expense of overtreating a proportion of men and accepting the attendant risk of treatment-related morbidity. Aggressive and radical treatment of a disease with a high incidence-to-mortality ratio would be widely accepted as reasonable and appropriate if the treatment had few side effects and was well tolerated. However, all primary treatments of prostate cancer have the potential to produce undesirable side effects in a substantial portion of men.

## CHARACTERISTICS OF LOCALIZED PROSTATE CANCER

### Unifocal vs. Multifocal

Prostate cancer has been shown to be multifocal. Even when the cancer is multifocal, most nonindex tumors appear to be biologically indolent based on their small size and low grade. Rukstalis et al. analyzed 112 whole-mount prostatectomy specimens and found 20% to have a unifocal cancer[11]. Among multifocal cancers, the index lesion had a median cancer volume of 1.6 $cm^3$, the median volume of each additional cancer was 0.3 $cm^3$, 80% of ancillary foci were <1.0 $cm^3$, and the mean total volume of all nonindex cancers was 1.2 $cm^3$. Ohori et al. analyzed 1,832 radical prostatectomy specimens by a whole-mount technique and showed the mean volume of the five largest cancers to be 2.13, 0.39, 0.17, 0.09, and 0.04 $cm^3$, respectively[12]. Among patients with multifocal disease, 80% of the total tumor volume was present in the index tumor. Extraprostatic extension arose from the largest cancer in 92% of patients. Of patients at low risk, defined as clinical stage T1c/T2a or less, biopsy Gleason score ≤6 and PSA <10 ng/ml, 28% had a unifocal tumor and only 1% had extraprostatic extension at the site of a secondary tumor.

### Predicting Biologic Indolence

Numerous studies have retrospectively sought to identify patients with indolent cancer based on pretreatment parameters. Cheng et al. found the highest percentage of cancer at any biopsy site and the total number of positive biopsy sites to predict the likelihood of small-volume cancer (<0.5 cc) on final pathologic evaluation[13]. For instance, in men with ≤5% of cancer at a single biopsy site, 30% had small-volume cancer. Among the 55 patients with small-volume cancer, 37 (67%) had multifocal cancer, highlighting that low tumor volume does not necessarily imply unifocality.

Epstein et al. used PSA density and pathologic findings on biopsy to predict insignificant cancers (defined as tumor volume <0.2 $cm^3$, Gleason <7, and confined to the prostate) with 73% accuracy[14]. Similarly, Goto et al. analyzed 170 prostatectomy specimens and found that among 12 patients with a PSA density <0.1 ng/ml/ml and maximum biopsy cancer length <2 mm, 9 (75%) met criteria for a clinically unimportant cancer (defined as tumor volume <0.5 $cm^3$, Gleason <7, and confined to the prostate)[15].

Kattan et al. incorporated PSA, clinical stage, prostate volume, Gleason score, total length of cancer on biopsy, and total length of noncancer on biopsy to construct a nomogram predicting indolent cancer (defined as <0.5 $cm^3$, moderately differentiated with no Gleason component of 4 or 5, and confined to the prostate) with excellent discriminatory ability and calibration[16]. This nomogram has been independently validated in a cohort of patients from the screened arm of the European Randomized Study of Screening for Prostate Cancer (ERSPC)[17].

## PRETREATMENT CANCER CHARACTERIZATION

### Magnetic Resonance Imaging (MRI)

In order to identify candidates for focal therapy properly and confidently, it is imperative to have a reliable means for localizing and accurately delineating the tumor. Imaging would ideally provide an acceptable standard by which prostate cancers could be identified and characterized. In addition to patient selection, accurate imaging modalities would prove useful in directing therapy, assessing results, and monitoring for disease recurrence or progression. The ability to assess locoregional lymph nodes adequately to evaluate for metastatic spread is also needed. Among currently utilized prostate imaging modalities (ultrasound, computed tomography, and MRI), endorectal MRI with spectroscopy (MRSI) appears to offer the best operating characteristics and enhances the predictive accuracy of standard nomograms.

The combination of endorectal MRI/MRSI and a clinicopathologic nomogram, when compared to the nomogram alone, adds substantially to the area under the receiver operating characteristic (ROC) curve (AUC) for predicting organ-confined cancer[18], extraprostatic extension[19], seminal vesicle invasion[20], and lymph node metastases[18].

MRI can also assist in the estimation of tumor volume. In a study by Coakley et al.[21], the accuracy of MRI and MRSI in the measurement of prostate cancer tumor volume was assessed in 37 patients prior to radical prostatectomy. For peripheral zone tumor nodules >0.5 $cm^3$, tumor volume measurements by MRI, MRSI, and combined MRI/MRSI were all positively correlated with histopathologic findings, but only measurements by MRSI and combined MRI/MRSI reached statistical significance. These results show that the combination of MRSI and MRI increases the overall accuracy of tumor volume measurement for lesions of this size, although measurement variability may limit consistent quantitative estimation in smaller tumors.

While MRI augments standard clinical and pathologic parameters in predicting advanced disease features and tumor volume, it is unable to visualize reliably the small and well-differentiated cancers that may be the prime candidates for either active surveillance or focal therapy. When it occasionally does highlight the area of a small, unifocal tumor, image-guided focal ablation may confidently target that region. However, the clinical utility of MRI rests as much, if not more, on what it does not show. Imaging that suggests the absence of a cancer that is large, poorly differentiated, or has pathologically advanced features increases support for the diagnosis of a small cancer with little risk. Additionally, the intended role of MRI would be to guide focal ablative therapy more precisely since it provides a very detailed view of the normal prostate overall and can help target the proper region (Fig. 1). Finally, MRI may be useful in monitoring the response to therapy by visualizing the size and location of the ablated prostate.

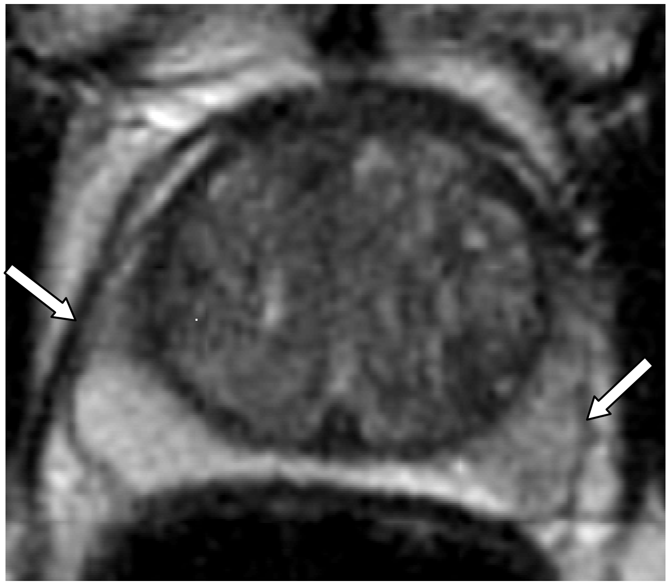

**FIGURE 1.** Axial T2-weighted MRI of the prostate with endorectal coil. Arrows indicate regions positive for prostate adenocarcinoma on biopsy. (Courtesy of Doug Pendsé, MD, Hashim Uddim Ahmed, MD, and Mark Emberton, MD.)

### Biopsy Characterization Strategies

Prostate biopsies have evolved from finger-directed to ultrasound-guided, usually with injection of an anesthetic and commonly sampling a larger portion of the prostate. Current approaches call for 10–12 biopsy cores to be taken, resulting in increased detection of tumors[22,23]. Extended biopsy schemes (>6 cores) can achieve a more thorough and accurate characterization of prostate tumors. In patients undergoing extended biopsies, the rate of Gleason upgrading on pathologic analysis of the prostatectomy specimen decreases[24,25]. Certainly transrectal biopsy strategies do not reliably identify all tumors present in the gland, leaving the clinician unaware of multifocal tumors in many patients. Multiple strategies have been employed in an attempt to characterize maximally tumor volume, location, and Gleason grade. Transperineal extended mapping biopsies have the potential to provide more detailed spatial and histologic information, but are more invasive. They require anesthesia and are associated with a greater risk of side effects, such as urinary retention[26,27]. As an alternate strategy, repeat extended transrectal biopsy may also be effective in identifying more extensive or higher-grade cancer in a subset of patients. With either approach, the absence of cancer or findings similar to the initial biopsy can reaffirm the likelihood of low-volume, well-differentiated cancer. The optimal technique of early repeat biopsy for patients considering active surveillance or focal therapy remains to be established, although the role for more thorough biopsy assessment prior to initiating these approaches seems clear.

## RATIONALE FOR FOCAL THERAPY

Historically, definitive treatment of solid cancers included whole-gland extirpation with radical surgery. For many organ systems, outcome data have supported more selective, organ-sparing, or even ablative therapies, such as breast-conservation surgery and less extensive surgery for melanoma[28,29].

Similar trends have been witnessed in urologic oncology. Historically, the standard treatment for all solid renal masses was radical nephrectomy. Within the past decade, cancer control following partial nephrectomy has been shown to be equivalent for tumors <4 cm, with the benefit of preserving renal function[30,31]. Once established as an accepted treatment for smaller tumors, investigators have subsequently shown similar outcomes following partial and radical nephrectomy in patients with masses ≤7 cm[32]. Likewise, partial cystectomy has proven effective for the treatment of bladder cancer in appropriately selected patients.

Prostate cancer may be amenable to organ-sparing, focal treatment. The prostate is a small, easily accessible organ, and many urologists are familiar with performing image-guided procedures in the gland through the rectum, perineum, or urethra. Intuitively, treatment would be required solely at the area of tumor, limiting collateral damage to normal tissue. While the potential of such therapies makes them attractive, the ramifications of treatment failure bear considerable forethought in the development of focal treatment trials. The issues of patient selection, appropriate targeting of lesions, and the impact of focal treatments on outcome with salvage surgical or radiation treatment all deserve consideration. Criteria for follow-up and patient evaluation, as well as indications for repeat treatment, also remain to be standardized.

The role for focal therapy in the treatment of prostate cancer may be most applicable to patients with tumors that pose little risk of progression, as long as the treatment has minimal effect on quality of life and does not adversely impact survival. Focal ablation of the index cancer, or of the sector of the prostate that harbors that cancer, could be very attractive for patients with low-risk cancers who are uncomfortable with the risks associated with active surveillance and the side effects of radical therapy. The unanswered questions are whether appropriate candidates can be identified, whether the index cancer can be reliably characterized while excluding higher-grade secondary tumors, and whether focal ablation can reliably target and destroy the index cancer with few complications and side effects. Even with effective treatment, such patients will have to be followed closely after focal therapy, since they may be at high risk for developing another cancer in the prostate. Ultimately, the benefits of focal therapy will need to be proven with a randomized clinical trial comparing focal ablation to active surveillance or radical therapy.

With these considerations in mind, a patient best suited for focal therapy would have a well-localized, targetable tumor of relatively small volume with a low-to-intermediate risk of metastatic spread and compliant with follow-up including repeat prostate biopsy procedures. Treatment must reliably eradicate tumors, have a low rate of side effects, and not complicate management with other therapies that may become necessary. One option for focal treatment that appears to meet several of these criteria is photodynamic therapy, which involves athermic treatment with relative sparing of tissue outside of the treatment field.

## PHOTODYNAMIC THERAPY

### Background

Photodynamic therapy (PDT) is an ablative therapy that depends on three elements: a photosensitizer, light, and oxygen. The photosensitizer is typically given systemically by intravenous infusion and is present in the tissue of interest by perfusion of that organ. The photosensitizer is activated at the intended site of action, typically by fiberoptic illumination at a wavelength matched to the properties of the drug. In tissues, lesion size is dependent on a variety of factors, including tissue vascularity, drug concentration, and light intensity and duration.

Through an unknown mechanism, photosensitizers preferentially accumulate in regions of proliferating cells, allowing PDT to display some selectivity for targeting a tumor while minimizing damage to surrounding normal tissue. The light-sensitive photosensitizing agent, which may be administered either topically or intravenously, is activated by a specific wavelength of light energy and creates oxygen-dependent cytotoxic and vasculotoxic reactions[33]. Subsequent loss of endothelial integrity leads to vascular thrombosis and ultimately tumor ablation[34,35,36]. Photosensitizers that have been studied include hematoporphyrin derivatives (Photofrin), m-tetrahydroxyphenylchlorin (Foscan), etiopurpurin dichloride (PhotoPoint), lutetium texaphyrin (LuTex), verteporfin (Visudyne), and palladium bacteriopheophorbide (WST09, also known as Tookad).

Current PDT techniques primarily target the cellular compartment to destroy a tumor, but newer vascular-targeting photosensitizers (verteporfin, WST09) are activated while in the vasculature. This property can be exploited to cause vascular damage (occlusion, perforation, and stasis), enhancing tissue necrosis and allowing improved penetration of cytotoxic agents[35,37,38,39,40]. Verteporfin has been primarily assessed in preclinical models[41,42], while WST09 has been assessed in both the preclinical setting and early human trials.

Given the ease of photosensitizer application, PDT is most frequently used on cutaneous lesions, but has also been tested on breast, central nervous system, head and neck, lung, esophageal, cervical, bladder, and prostate cancers. When used as prostate cancer therapy, advantages of PDT include minimally invasive access via the perineum, with techniques similar to brachytherapy (Fig. 2), and the ability to retreat lesions if necessary. Lesions created with PDT can be well visualized with perfusion imaging including gadolinium-enhanced endorectal MRI (Fig. 3). Disadvantages include risks of cutaneous and ocular photosensitivity, localized collateral damage to adjacent organs (including bowel, urethra, bladder, and nerve tissue), and systemic toxicity associated with intravenous administration that may include vasculopathy or involvement of other organs.

### Preclinical Studies

WST09 is one of the latest PDT agents developed through the Weizmann Institute in Israel. WST09 is a chlorophyll-derived photosensitizer with several desirable properties, including rapid intravascular clearance

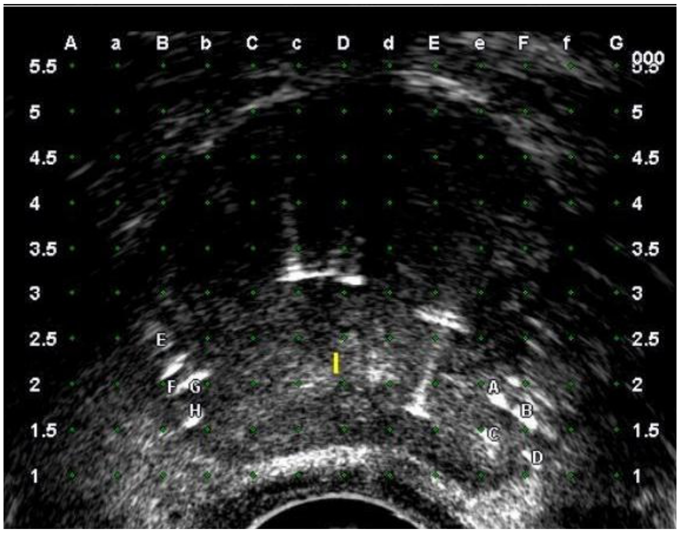

**FIGURE 2.** Axial transrectal ultrasound image of mid-prostate with fiberoptic fibers after positioning with stereotactic brachytherapy equipment for focal treatment. Fibers are labeled A–D in right lobe and E–H in left lobe. The urethra is indicated by a small vertical yellow line. Other echoic features indicate location of optical monitoring sensors. (Courtesy of Doug Pendsé, MD, Hashim Uddim Ahmed, MD, and Mark Emberton, MD.)

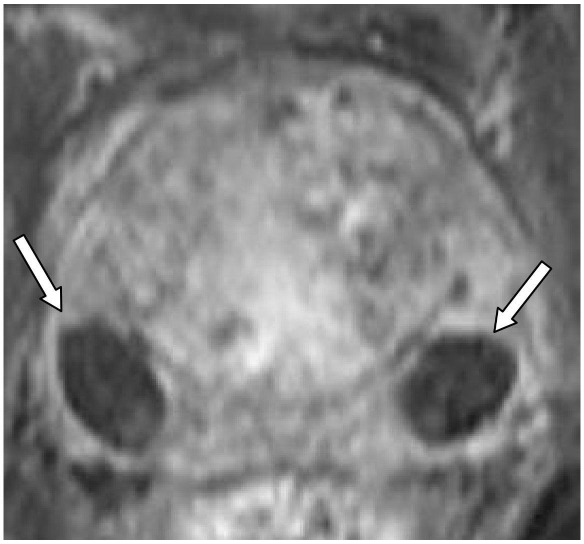

**FIGURE 3.** Axial T1, postgadolinium-enhanced MRI of the prostate after focal treatment. The dark, low-signal areas (indicated by arrows) demonstrate lack of perfusion in the zone of treatment. (Courtesy of Doug Pendsé, MD, Hashim Uddim Ahmed, MD, and Mark Emberton, MD.)

and minimal tissue accumulation, decreasing the risks of photosensitivity. Its absorption wavelength at 763 nm is within the near infrared range, allowing reasonable tissue penetration using fiberoptic light application. Preclinical studies in animal models have demonstrated its safety profile and antitumor effect.

Initial investigations in both canine and murine models identified the safety and treatment parameters for WST09-PDT treatment. Intravenous drug infusion to a dose concentration between 2 and 4 mg/kg was well tolerated. Fiberoptic-delivered interstitial illumination with a diode laser at lambda = 770 nm was capable of creating lesions of hemorrhagic necrosis as large as 3 cm in the canine prostate gland[43]. Heterotopic and orthotopic tumors created with WISH-PC2 cells in the murine model were effectively eradicated in over 50% of lesions followed over 70 days[35].

Photosensitivity studies were performed in both animals and humans to evaluate any cutaneous reaction to incident full-spectrum light exposure. Drug dosing at 2 mg/kg and skin exposure to both UV- and UV+ solar-simulated light at 128 J/cm$^2$ did not demonstrate skin photosensitivity at exposure durations up to 3 h. Drug doses >2 mg/kg were associated with evidence of phototoxicity in the murine and porcine models, and were not administered to human subjects[44].

Damage to tissue structures outside of the prostate gland are possible with PDT therapy and are likely when illumination outside of the boundaries of the prostate occurs. Evaluation of tissue sensitivities to WST09-PDT was performed in the canine model. Nerve conduction studies immediately and 1 week following treatment to the cutaneous branch of the saphenous nerve revealed light dose-dependent functional damage when light exposure over 100 J/cm$^2$ was used. Exposure at 50 J/cm$^2$ showed minimal conduction effects[45]. Direct treatment to regions outside the prostate, including the colon, bladder, abdominal muscle, and pelvic plexus, showed treatment effects consistent with necrosis in these areas. However, reaction within the adjacent prostate was considered more extensive on histologic analysis, suggesting some degree of tissue selectivity[46].

## WST09-PDT Technique

A feasibility study of PDT in humans has been completed. In a Phase I trial of 24 patients with radiorecurrent prostate cancer, dose escalation of WST09 up to 2 mg/kg and light doses up to 360 J/cm were generally well tolerated[47]. Intravenous infusion of the photosensitizer occurred over a 20-min period. Since a proportion of patients experienced transient hypotension during the infusion, all patients were premedicated with an antihistamine and given intravenous fluid or vasopressors, as needed. In this published study, no serious adverse events were noted, including skin photosensitivity.

Using general anesthesia, the patient was positioned in the lithotomy position with an ultrasound probe in the rectum and a brachytherapy template applied to the perineum. A catheter was inserted into each lobe of the prostate via the template and visualized via the transrectal probe. Optical fibers set at 763 nm for light delivery and probes to detect light dosimetry and temperature were placed within the catheters. Additional probes were placed within a translucent urethral catheter and the anterior rectum to monitor light exposure to these areas. To minimize rectal exposure to light, a hydrodissection procedure on the plane between the rectum and prostate was performed. Following infusion of WST09, escalating doses and duration of light were administered. No signs of treatment response were seen at a drug dose <1.0 mg/kg, leading investigators to define these patients as nonresponders in the trial.

In addition to safety data collected as part of this Phase I trial, tissue end points were also evaluated by interval radiographic imaging and prostate biopsy. At 7 days following treatment, hypoperfusion was noted on MRI at the maximum drug and light doses. Biopsies performed 6 months after treatment showed fibrosis and no evidence of viable cancer in areas found to be avascular on post-treatment MRI in men with response. However, small islands of benign tissue were seen, possibly indicating a selective effect of the treatment on tumor-bearing tissue. Treatment effects were not evident on biopsies from untreated regions. PSA declined to negligible levels in four of the six patients who received the maximum light and photosensitizer dose. Evidence of transient hepatic enzyme increase was seen in a subset of patients without sequelae.

All patients voided spontaneously following 7–14 days of catheterization. A significant decline in urinary function was noted in five patients who demonstrated treatment response, defined by a prostate necrosis volume ≥20% on MRI 7 days post-PDT. The mean change in International Prostate Symptom Score (IPSS) score at 1 month following treatment was 20 points with gradual improvement resulting over the subsequent 5 months. Bowel and erectile function were not significantly affected, as measured by the Patient-Oriented Prostate Utility Scale (PORPUS)[48].

Mature data from this trial and others are required to assess the potential role of PDT in treating localized prostate cancer following radiation therapy. Given the encouraging safety profile and evidence of a treatment effect, a Phase II trial of 24 patients in a similar clinical setting has begun. Of the 16 patients with ≥6 month follow-up in the Phase II trial, hypoperfused MRI lesions and decreases in PSA have been noted. Given the results of these preliminary trials, PDT is also actively being investigated for primary treatment of low-risk, localized prostate cancer.

## RECOMMENDATIONS FOR RESEARCH OF PDT AND OTHER POTENTIAL FOCAL THERAPIES

Single-arm feasibility trials with PDT have been designed as acceptable initial investigations in focal therapy. Further multiarm studies using several techniques for localized therapy will ideally follow. Critical to the advancement of focal treatment will be appropriate trial design and careful comparative assessment to identify superiority in technique and technology, as well as allow for continued advancements in the field. Unfortunately, as experienced with several other experimental ablative procedures in cancer care, exuberant enthusiasm should not supersede proper investigation and introduction of these techniques into clinical care. Outcome measures for focal therapy trials should include (a) scheduled systematic post-treatment mapping biopsy to determine the boundaries of the treatment effect and to assess for recurrent or new disease, (b) disease-free progression, and (c) morbidity outcomes and longitudinal health-related quality of life measures. Eventually, a rigorous comparison must be made between patients treated with focal ablation, and an age- and comorbidity-matched population of patients undergoing expectant management or an established treatment intervention.

## ACKNOWLEDGMENTS

This work was supported by a grant from the National Cancer Institute (P50-CA92629 SPORE) and philanthropic grants from the Skirball Foundation and the Lowenstein Foundation. Scott E. Eggener was funded through a National Institute of Health Ruth Kirchstein National Research Service Award (T32-CA82088-06). The authors are grateful to Doug Pendsé, MD, Hashim Uddim Ahmed, MD, and Mark Emberton, MD for permission to use their images, and to Michael McGregor for assistance in preparing the manuscript.
